# Homophily, Close Friendship, and Life Satisfaction among Gay, Lesbian, Heterosexual, and Bisexual Men and Women

**DOI:** 10.1371/journal.pone.0128900

**Published:** 2015-06-18

**Authors:** Brian Joseph Gillespie, David Frederick, Lexi Harari, Christian Grov

**Affiliations:** 1 Department of Sociology, Sonoma State University, Rohnert Park, California, United States of America; 2 Crean School of Health and Life Sciences, Chapman University, Orange, California, United States of America; 3 Department of Sociology, California State University, Channel Islands, Camarillo, California, United States of America; 4 Department of Health and Nutrition Sciences, Brooklyn College, Brooklyn, New York, United States of America; University of Toronto, CANADA

## Abstract

Friends play important roles throughout our lives by providing expressive, instrumental, and companionate support. We examined sexual orientation, gender, and age differences in the number of friends people can rely on for expressive, instrumental, and companionate support. Additionally, we examined the extent to which people relied on same-gender versus cross-gender friends for these types of support. Participants (*N* = 25,185) completed a survey via a popular news website. Sexual orientation differences in number of same-gender and cross-gender friends were generally small or non-existent, and satisfaction with friends was equally important to overall life satisfaction for all groups. However, the extent to which people’s friendship patterns demonstrated gender-based homophily varied by sexual orientation, gender, and age. Young adult gay and bisexual men, and to some extent bisexual women and older bisexual men, did not conform to gendered expectations that people affiliate primarily with their own gender.

## Introduction

Friends play a critical role in enhancing life satisfaction and psychological well-being [1]. The goal of this study was to investigate whether sexual orientation was related to friendship patterns in a diverse U.S. online study. We examined whether gay, lesbian, and bisexual (GLB) individuals reported more friends than heterosexuals and whether they were less likely than heterosexuals to engage in friendships defined by gender homophily (i.e., the tendency to associate with individuals of the same gender). Finally, given the importance of social support for individuals at risk for experiencing “minority stress” (the negative symptoms, such as depression and suicidal thoughts, that GLB individuals are likely to experience as a result of their stigmatized identity) and rejection from peers and parents [2], we examined whether friends were a more important source of overall life satisfaction for GLB individuals. Thus, we examine the extent to which GLB individuals conform to traditional gendered expectations for close friendships. We also explore whether these friendships are more important for GLB individuals than their heterosexual counterparts.

### Sexual Orientation Differences in Friendship Patterns

The first objective of this research was to examine if sexual orientation was associated with number of friends. There has been an upsurge in research on how close friendship relates to the well-being of gay men and lesbians, while bisexual friendship research remains conspicuously absent [3–4]. Much of this research has emphasized the importance of close friendships for gay men and lesbians because they are at a greater risk for developing adverse mental health outcomes compared to their heterosexual counterparts [2, 5] and friendship may mitigate risks to mental health [6–7].

Gay men and lesbians in particular receive more substantial social support from friends than heterosexual men and women [5, 8]. The importance of friendship among lesbians, gay men, and bisexual men and women can be substantiated by minority stress theory [2]. Minority stress theory proposes that GLB individuals are likely to experience rejection as a result of their marginalized sexual orientation and must learn to cope and adapt to a potentially “inhospitable social environment” [9]. Since prior research indicates that close friendships help GLB individuals foster a sense of identity [10–12], it is understandable that sexual minorities may not only want more friends, but *need* more friends (i.e., individuals who affirm their minority identity) compared to their heterosexual counterparts.

Some research has suggested that gay and lesbian individuals have more friends than heterosexuals [13], but less is known about bisexuals. It is possible that bisexual individuals report *fewer* friends compared to their gay, lesbian, and heterosexual counterparts due to “biphobia.” Biphobia implies that bisexual individuals endure a unique situation regarding friendships, given that many gay men, lesbians, and heterosexual men and women regard the bisexual identity as an unstable one compared to more “legitimate” identities—the dichotomous categories of “gay” and “straight” [4, 10]. Because of this stigmatizing nature of biphobia, bisexual men and women may find it more difficult to form and maintain friendship ties.

In this study, we focus on three types of friendship: expressive, instrumental, and companionate. Much past research has simply asked people how many friends they have [14], but here we wanted to explore specific operationalizations of these friendship types. In terms of instrumental support, research has suggested that men’s friendships are largely defined by doing things for people they care about and that availability of tangible help is a defining feature of friendship [15–16]. Therefore, we asked respondents how many people they could call or text if they found themselves in trouble late at night.

Regarding expressive support, some research has suggested that women’s friendships revolve around emotional expressiveness and the disclosure of intimate information [17]. We wanted to explore a specific aspect of expressiveness, however, that may differ from typical gendered patterns: discussing one’s sex life. Discussion of personal matters with friends, specifically sex-related topics, is characteristic of close and emotionally intimate friendships [18]. It is unknown whether GLB individuals are more comfortable discussing their sex lives than heterosexual individuals. Sexual minorities have far fewer positive media representations of same-gender sexuality [19], and thus relying on friends for discussions of sexuality may take on greater importance.

Recent research has expanded the instrumental-expressive framework to include the importance of engaging in shared activities, or “companionate” friendship [1]. Since research indicates that the celebration of birthdays is especially important among women [20], and that sharing positive events is an important component of companionate close friendship [1], we asked respondents how many people they would “expect to do something” with them to celebrate their birthday.

### Gender Homophily by Sexual Orientation

The second objective of our study was to examine if gender homophily—a well-established phenomenon in heterosexual friendships—exists to a lesser degree among GLB men and women. Homophily is the notion that people affiliate with others who are similar to themselves [21], and homophily in the form of same-gender relationships may be common among heterosexuals in part because cross-gender friendships can add the complicating factor of possible romantic or sexual tension or the jealousy of one’s partner [22–23], which may be less common among gay men and lesbians. Gay men and lesbians already do not conform to traditional gendered expectations for romantic relationships, and may be more comfortable transgressing against gendered expectations for friendship [24].

There is a stereotype that gay men and heterosexual women frequently pair together for friendships and this is possible, in part, due to the supposed asexual nature of these friendships [25–26]. Heterosexual women tend to express less prejudice than heterosexual men towards gay men [27], and thus cross-gender friendship for gay men may provide an opportunity for a larger social support network. Consistent with this proposal, past research has found that heterosexual women are more likely than heterosexual men to report having gay male friends who have come out to them [28].

### Friendship and Well-Being

Having a wide network of close confidants to affirm one’s identity may offset the adverse effects of minority stress, such as dealing with homophobic attitudes and discriminatory social structures and norms [29]. Although Meyer’s [2] theory emphasized friendships with other GLB individuals as an effective coping mechanism for minority stress for GLB individuals, we expand on this premise by including friendships and social support more generally. Indeed, Meyer [2] notes that any group solidarity can contribute to positive mental health. Therefore, the third objective of this research was to test, within the theoretical frameworks of minority stress theory, whether the quality and/or quantity of close friendships are more strongly linked to overall life satisfaction for GLB men and women than for heterosexuals.

### Hypotheses

We derived the following hypotheses based on the existing literature:

**Hypothesis 1:** Sexual orientation would be associated with number of friends. We hypothesized that heterosexual participants would report fewer friends than gay men, lesbians, and bisexual men and women, but the effect size might be larger between heterosexual vs. GL than heterosexual vs. bisexual participants. We did not expect an interaction between gender and sexual orientation (i.e., we did not expect that differences in numbers of friends between GLB individuals would vary based on gender).

**Hypothesis 2:** Homophily would be more pronounced among heterosexual men and women than among GLB men and women.

**Hypothesis 3:** Friendship satisfaction and number of friends would be more strongly linked to life satisfaction for GLB men and women than for heterosexual men and women.

## Methods

### Participant Recruitment

The present study is based on secondary analyses of an anonymous survey posted on the official website of NBC News (formerly msnbc.com, now NBCNews.com) for ten days in April of 2010. As such, the present research was deemed exempt from Institutional Review Board review. Participants were volunteers who clicked on a banner advertisement for the “Sex, Stress, and Success” survey. The survey software denied multiple submissions from any given computer. All relevant data have been included as S1 Dataset.

Market research on NBCNews.com shows that at the time of the survey it routinely ranked among one of the most popular non-pornographic websites in the United States. Its 58 million unique monthly visitors include a broad diversity of people in the country in terms of age, income, and political orientation [30]. Note that msnbc.com, the general news website, was a different entity than MSNBC TV and had substantially different demographics (e.g., msnbc.com has more equal numbers of Democrat and Republican visitors). Data garnered through this site have been used successfully for various examinations of elements of body dissatisfaction, sexuality, and close relationships [31–33].

### Sample Characteristics and Variables

Data on the number of individuals who clicked the link to start the survey but did not complete were not available to the research team. The 25,185 participants who completed the survey consisted of 11,924, heterosexual men, 387 bisexual men, 343 gay men, 220 lesbian women, 511 bisexual women, and 11,800 heterosexual women. The average *age* for the sample was 42.3 (*SD* = 13.1). Variation in age and other sample characteristics by sexual orientation are presented in Table 1.

**Table 1:** Sample Characteristics (*N* = 25,185).

	Lesbian Women(*n* = 220)	Bisexual Women(*n* = 511)	Heterosexual Women(*n* = 11,180)	Gay Men(*n* = 343)	Bisexual Men(*n* = 387)	Heterosexual Men(*n* = 11924)	Analysis
	*M* (*SD*)	*M* (*SD*)	*M* (*SD*)	*M* (*SD*)	*M* (*SD*)	*M* (*SD*)	*F* (*df*)
Age (18–75)	41.4 (12.8)	33.3 (11.3)	38.6 (12.2)	40.8 (13.0)	46.7 (13.9)	46.3 (12.8)	509***
Income (2.5K–1500K)	71K (145K)	48K (119K)	54K (75K)	72K (74K)	100K (188K)	108K (149K)	251***
	% (*n)*	% (*n)*	% (*n)*	% (*n)*	% (*n)*	% (*n)*	
High school or less	7 (15)^a^	10 (50)^b^	8 (909)^c^	6 (20)^ac^	10 (39)^abc^	8 (911)^ab^	126***
Some college or A.A.	29 (64)	41 (209)	33 (3914)	32 (110)	34 (132)	29 (3466)	
College degree	26 (57)	23 (120)	32 (3766)	29 (101)	27 (106)	31 (3736)	
Advanced degree	38 (84)	26 (132)	27 (3211)	33 (112)	29 (110)	32 (3811)	
Married	39 (86)^a^	42 (217)^a^	50 (5861)^b^	31 (107)^a^	66 (254)^a^	72 (8535)^c^	1508***
Not dating	21 (46)	15 (74)	17 (1989)	34 (115)	15 (59)	10 (1218)	
Casually dating	11 (24)	9 (48)	7 (898)	10 (36)	8 (32)	5 (586)	
In relationship	29 (64)	34 (172)	26 (3052)	25 (85)	11 (42)	13 (1585)	
No children	71 (157)^a^	59 (301)^b^	46 (5423)^c^	86 (294)^d^	31 (121)^c^	25 (2932)^f^	1771***
Children	29 (63)	41 (210)	54 (6377)	14 (49)	69 (266)	75 (8992)	
	*M* (*SD*)	*M* (*SD*)	*M* (*SD*)	*M* (*SD*)	*M* (*SD*)	*M* (*SD*)	*F* (*df*)
*Satisfaction*							
Life	5.47 (1.36)^ac^	5.22 (1.42)^bc^	5.45 (1.33)^a^	5.07 (1.47)^b^	5.18 (1.50)^bc^	5.42 (1.39)^a^	9.7***
Relationship w/ friend	5.31 (1.58)^abc^	5.13 (1.58)^b^	5.39 (1.45)^a^	5.27 (1.67)^abc^	4.98 (1.67)^b^	5.28 (1.42)^c^	13.8***
Job	4.83 (1.88)^ab^	4.48 (1.98)^b^	4.71 (1.89)^b^	4.51 (1.90)^b^	4.83 (1.91)^ab^	4.95 (1.81)^a^	24.4***
Health	5.18 (1.53)^ab^	4.93 (1.63)^b^	5.26 (1.47)^a^	5.06 (1.49)^ab^	4.90 (1.66)^b^	5.27 (1.44)^a^	10.9***
Physical Appearance	4.39 (1.76)^a^	4.56 (1.71)^ab^	4.55 (1.60)^a^	4.53 (1.54)^ab^	4.54 (1.61)^a^	4.77 (1.47)^b^	28.0***

*** *p* < .001.

All reported pairwise comparisons are significant at the *p* < .01 level using Games-Howell post-hoc or Chi-Square analyses as appropriate. Common superscripts indicate non-significant differences. For the satisfaction measures, 1 = Very dissatisfied, 4 = Neutral, 7 = Very satisfied.

#### Number of friends and expressive, instrumental, and companionate attributes of friendship

*Same-gender* and *cross-gender* friendships were assessed through three items. Participants were presented with the question stem “Thinking of your female and male friends, how many do you have that…” with three items to which they responded: “You could talk to about your sex life?” (*Expressive*), “You could call/text if you were in trouble late at night?” (*Instrumental*), and “You expect to do something with you to celebrate your birthday?” (*Companionate*). For each of these three items, participants were presented with a drop-down menu for “number of female friends” and “number of male friends” separately with the following responses for each: 0, 1, 2, 3–5, 6–10, 11–15, 16–20, 21+. For the options with ranges, the participant was given the midpoint score (e.g., a score of 4 was assigned for the 3–5 category) and 21+ was assigned a code of 23.

#### Homophily measures

We calculated homophily for friendship type (*expressive*, *instrumental*, *companionate*) by taking the number of same-gender friends for each participant and subtracting the number of cross-gender friends for each participant. For example, if a participant had 5 same-gender friends and 3 cross-gender friends on whom they could count to celebrate their birthday, their birthday homophily score would be a “2.”

#### Sociodemographic variables

*Income* was based on the logged measure of the respondent’s reported level of personal income. A total of 18 response categories ranged from 0-$4,999 at the lower end through $1 million or more at the upper end. The variable was recoded using the midpoint value for each income category, and the amount of $1,000,001 was used as the highest income. The overall median income was $55,000.

*Educational status* was an ordinal measure. For regression analyses, the ordinal categories were coded 0–3 to indicate lower versus higher levels of education, coded as high school or less (0), some college or associate’s (1), college degree (2), and advanced degree (3).

*Marital status* was assessed using a dichotomous variable marking whether an individual was not married (Men = 30 percent, Women = 51 percent) versus married, in a civil union, or in a legalized domestic partnership. In regression analyses, nonmarried individuals were coded as 0 and married individuals were coded as 1. *Children* were reported by 63 percent of the sample (Men = 74 percent, Women = 53 percent). For regression analyses, respondents without children were coded as 0 and those with children were coded as 1.

#### Satisfaction measures

Participants were presented with the following question stem “On a scale from 1–7, how dissatisfied or satisfied are you with your…” and then a randomized series of items, including: “Life overall,” “Current job situation,” “Physical appearance,” “Health,” and “Relationships with your friends.” The scale ranged from *very dissatisfied* (1) to *neutral* (4) to *very satisfied* (7), with the option to mark “not applicable.”

## Results

### Statistical Significance and Effect Size

Our large sample size enabled us to detect even miniscule effects when using the full sample of participants. For all associations, we highlight whether the results were statistically significant at the *p* < .001, .01, or .05 levels. Even with the most stringent criteria, Beta (*β)* values as small as .02 were statistically significant when using the full samples of men or women. Therefore, in addition to reporting statistical significance, we also attend to effect size. There are established rough guidelines for interpreting Cohen’s *d* effect sizes as small (.20), moderate (.50), or large (.80) [34]. To our knowledge, there are no such guidelines for interpreting *β* values. A *β* value of .10 indicates that as one increases one unit in the predictor variable, there is a corresponding .10 standard deviation increase in the outcome variable (controlling for other variables in the model). The predictors can either be categorical variables (e.g., male = 0 to female = 1) or z-scored continuous variables (e.g., from average = 0 to one standard deviation above the mean in age = 1). In this study, consistent with past research using these large data sets [31, 33], we elected to highlight statistically significant results in the text when they reflect *β* values greater than |.09| and Cohen’s *d* greater than |.19|, although we mark all significant associations in the tables.

### Hypothesis 1: Sexual Orientation Differences in Friendship Patterns

To test Hypothesis 1, that there would be sexual orientation differences in number of friends, we conducted a series of regression analyses with gender, sexual orientation, the interaction between gender and sexual orientation, demographic variables, and the two-way interactions between demographic variables with gender and with sexual orientation, for each of the friendship types (the full set of interactions were not conducted because they would have spliced the GLB participant groups into unacceptably small subsamples). Table 2 presents the test of Hypothesis 1, including interactions between gender and sexual orientation. Multicollinearity was not an issue in any analysis (VIFs were below 5.0 for all predictors for all analyses).

**Table 2:** Sexual Orientation and Number of Same-Gender and Cross-Gender Friends (Hypothesis 1).

	Friendship Type					
	Talk About Sex Life		Call At Night		Celebrate Birthday	
	Same-Gender	Cross-Gender	Same-Gender	Cross-Gender	Same-Gender	Cross-Gender
	*β*	*β*	*β*	*β*	*β*	*β*
Key Groups of Interest						
Bisexual	-.04*	-.03	-.02	-.02	-.02	.02
Gay/Lesbian	.01	-.02*	-.03*	-.02	.01	-.01
Gender (Male = 0)	-.02	-.17***	-.02	.04*	.10***	.03
Gender X Bisexual	.04***	.04**	.03**	.03*	.03**	.03*
Gender X Gay/Lesbian	-.01	-.01	.02*	-.02*	.00	-.01
Demographics						
Age	-.24***	-.07***	-.25***	-.09***	-.29***	-.15***
Age^2^ (curvilinear)	.07***	.00	.07***	.06***	.14***	.12***
Education	.00	-.04***	.05***	- .01	.04***	.01
Income	.05***	.02**	.05***	.04***	.06***	.06***
Children	-.01	.02	.01	.01	-.03*	-.01
Marital Status	-.15***	-.21***	.01	-.07***	-.04***	-.05***
Two-Way Interactions With Key Groups of Interest						
Age X Bisexual	.03**	-.01	.02**	.01	.03***	.02*
Age^2^ X Bisexual	.04***	.08***	.01	.03**	.00	.02
Marital X Bisexual	.01	.01	-.01	-.01	.01	.00
Children X Bisexual	.00	.02*	.00	.01	-.03*	-.02
Age X Gay/Lesbian	.03***	-.03***	.03***	-.01	.03***	.00
Age^2^ X Gay/Lesbian	.01	.05***	.02*	.03***	.01	.03**
Marital X Gay/Lesbian	.00	.02**	.00	.02**	.01	.01
Children X Gay/Lesbian	.01	.01	.01	.02*	.01	.02**
Age X Gender	.06***	.00	.11***	.01	.11***	-.01
Age^2^ X Gender	-.04***	.00	-.03*	.00	-.03**	-.01
Marital X Gender	.04**	.07***	-.02	.02	-.02	-.01
Child X Gender	.00	-.04**	-.01	-.04**	-.02	-.06***
Model Statistics						
*Adj*. *R* ^*2*^	.06***	.06***	.03***	.02***	.07***	.05***

*** *p* < .001

** *p* < .01

* *p* < .05.

Separate OLS regressions examining the predictors of number of same-gender and cross-gender friends are shown (e.g., the first column of beta values shows the predictors of number of same-gender friends with whom one can discuss their sex life). The reference groups for dummy coded variables were: Gender (Men), Gay/Lesbian and Bisexual (Heterosexual), Children (No Children), and Marital Status (Unmarried). The predictors were entered simultaneously.

As shown in Table 2, sexual orientation differences in number of friends were generally small, as were the interactions between gay men, lesbians, and bisexual men and women with the demographic variables (all *β*s < |.10|). Thus, in contrast to our hypotheses, there were no substantial differences between heterosexual participants and GLB participants when controlling for other variables.

### Hypothesis 2: Homophily

Overall, most groups showed some degree of homophily. Looking at average number of friends, most groups reported between 0.50 and 2.0 more same-gender friends than cross-gender friends across the different friendship questions (see top of Table 3).

**Table 3:** Same-Sex Homophily by Gender and Sexual Orientation (Hypothesis 2).

	Mean Number of Same-Gender minus Cross-Gender Friends					
	Heterosexual		Gay/Lesbian		Bisexual	
	Men	Women	Men	Women	Men	Women
	*M*	*M*	*M*	*M*	*M*	*M*
	(*SD*)	(*SD*)	(*SD*)	(*SD*)	(*SD*)	(*SD*)
All Participants						
Talk About Sex Life	.96	1.68	1.09	1.50	.29	1.08
	(2.87)	(2.23)	(3.89)	(2.92)	(3.67)	(3.29)
Call At Night If Trouble	1.48	1.08	.86	1.70	.96	.59
	(1.48)	(2.34)	(2.50)	(2.49)	(2.72)	(3.10)
Celebrate Birthday	.77	1.43	1.27	2.19	.11	.77
	(2.55)	(2.65)	(3.34)	(3.26)	(3.06)	(3.07)
	Comparison of Mean Number of Same- vs. Cross-Gender Friends					
	Heterosexual		Gay/Lesbian		Bisexual	
	Men	Women	Men	Women	Men	Women
	*d*	*d*	*d*	*d*	*d*	*d*
Participants Aged 18–29						
Talk About Sex Life	.57***	.81***	-.05	.56***	.08	.33***
Call At Night If Trouble	.68***	.39***	.00	.55***	.13	.19**
Celebrate Birthday	.58***	.45***	.14	.60***	-.07	.23***
Participants Aged 30+						
Talk About Sex Life	.30***	.73***	.47***	.50***	.08	.33***
Call At Night If Trouble	.53***	.50***	.53***	.82***	.43***	.19**
Celebrate Birthday	.25***	.58***	.44***	.71***	.08	.28***

*** *p* < .001

** *p* < .01.

Differences in mean number of same-gender versus cross-gender friends are displayed in the form of Cohen’s *d*. Since these comparisons all involved within-subjects comparisons, effect size *d* was calculated using Morris and DeShon’s equation 8 [43] rather than Cohen’s formula [34]. Statistical significance is based on the results of paired-samples *t*-tests. A positive effect size indicates that participants reported having more same-gender than cross-gender friends in that group. For example, heterosexual men ages 18–29 reported having more same-gender than cross-gender friends (*d* = .57).

To test Hypothesis 2 regarding sexual orientation differences in homophily, we conducted a series of regression analyses with each of the homophily measures as the outcome variable (Table 4). In these regressions, a positive beta value means that people scoring higher on predictor variables report relatively more same-gender than cross-gender friends than people scoring lower on the predictor variables. When looking at the sexual orientation comparisons, a positive beta would indicate that GLB participants reported more same-gender friends than heterosexual participants (reference group), whereas a negative beta would indicate that GLB participants reported fewer same-gender friends than heterosexual participants.

**Table 4:** Degree of Homophily by Gender, Sexual Orientation, and Demographics (Hypothesis 2).

	Degree of Homophily (number of same-sex minus cross-sex friends)								
	Full Sample			Men			Women		
	Talk Sex	Call at Night	Celebrate Birthday	Talk Sex	Call at Night	Celebrate Birthday	Talk Sex	Call at Night	Celebrate Birthday
	*β*	*β*	*β*	*β*	*β*	*β*	*β*	*β*	*β*
Key Groups									
Bisexual	-.02	-.01	-.05**	-.02	-.01	-.04	-.01	.00	-.03
Gay/Lesbian	.03*	-.02	.03*	.04**	.01	.06***	.01	.03	.02
Gender	.11***	-.06***	.11***	-	-	-	-	-	-
Gender X Bisexual	.02	.01	.02	-	-	-	-	-	-
Gender X Gay/Les	.00	.05***	.02*	-	-	-	-	-	-
Demographics									
Age	-.21***	-.22***	-.24***	-.21***	-.23***	-.27***	-.11***	.00	.03*
Age^2^ (curvilinear)	.08***	.04***	.07***	.10***	.06***	.10***	.01	-.02	.01
Education	.03***	.07***	.05***	.00	.03**	.01	.07***	.11***	.09***
Income	.03***	.02*	.02*	.06***	.07***	.06***	.01	-.03*	-.01
Children	-.03*	.01	-.02	-.03*	.00	-.02	.01	.04**	.02
Marital Status	.01	.08***	.00	.00	.07***	-.01	-.02	.03**	-.03**
Key Interactions									
Age X Bisexual	.03***	.02***	.02**	.05***	.05***	.06***	.01	-.02	-.02
Age^2^ X Bisexual	-.02*	-.02	-.01	-.03*	-.03*	-.03*	-.03	-.04**	-.03
Marital X Bisexual	.00	-.01	.02	.01	.01	.03	-.01	-.02	.01
Child. X Bisexual	-.02*	-.01	-.02	-.02	-.03	-.03	-.03	-.01	-.01
Age X Gay/Les.	.05***	.04***	.04***	.08***	.07***	.07***	.02	-.01	-.01
Age^2^ X Gay/Les.	-.03**	-.01	-.02*	-.06***	-.04**	-.05***	.01	.03*	.01
Marital X Gay/Les.	-.02***	-.02**	.00	-.02	-.03*	.00	-.03**	-.02	.01
Child. X Gay/Les	.00	.00	-.01	.01	.00	-.01	.00	.01	.01
Age X Gender	.07***	.13***	.17***	-	-	-	-	-	-
Age^2^ X Gender	-.05***	-.03***	-.04***	-	-	-	-	-	-
Marital X Gender	-.02	-.04***	-.02	-	-	-	-	-	-
Child. X Gender	.04*	.03	.03*	-	-	-	-	-	-
Model Statistics									
*Adj*. *R* ^*2*^	.04***	.03***	.04***	.04***	.04***	.06***	.02***	.02***	.01***

*** *p* < .001

** *p* < .01

* *p* < .05.

Separate OLS regressions examining the predictors of homophily are shown. Positive betas indicate that higher scores on the predictor are related to higher greater homophily (e.g., compared to men, women reported have relatively more same-sex friends than cross-sex friends with whom they could talk about their sex lives, *β* = .11). The reference groups for dummy coded variables were: Gender (Men), Gay/Lesbian and Bisexual (Heterosexual), Children (No Children), and Marital Status (Unmarried). The predictors were entered simultaneously.

At first glance, there did not appear to be large sexual orientation differences in degree of homophily (all *β*s < |.07|; Table 4). Looking at the main effect of sexual orientation, most comparisons of heterosexual to bisexual participants, and heterosexual to gay/lesbian participants, were not significant. The few that were significant were relatively small. There was a notable association of age with homophily, with homophily becoming less pronounced among older participants. Overall, women showed greater homophily than men for talking about sex life. There were a number of interactions, however, between age and sexual orientation and between age and gender.

**Fig 1:**
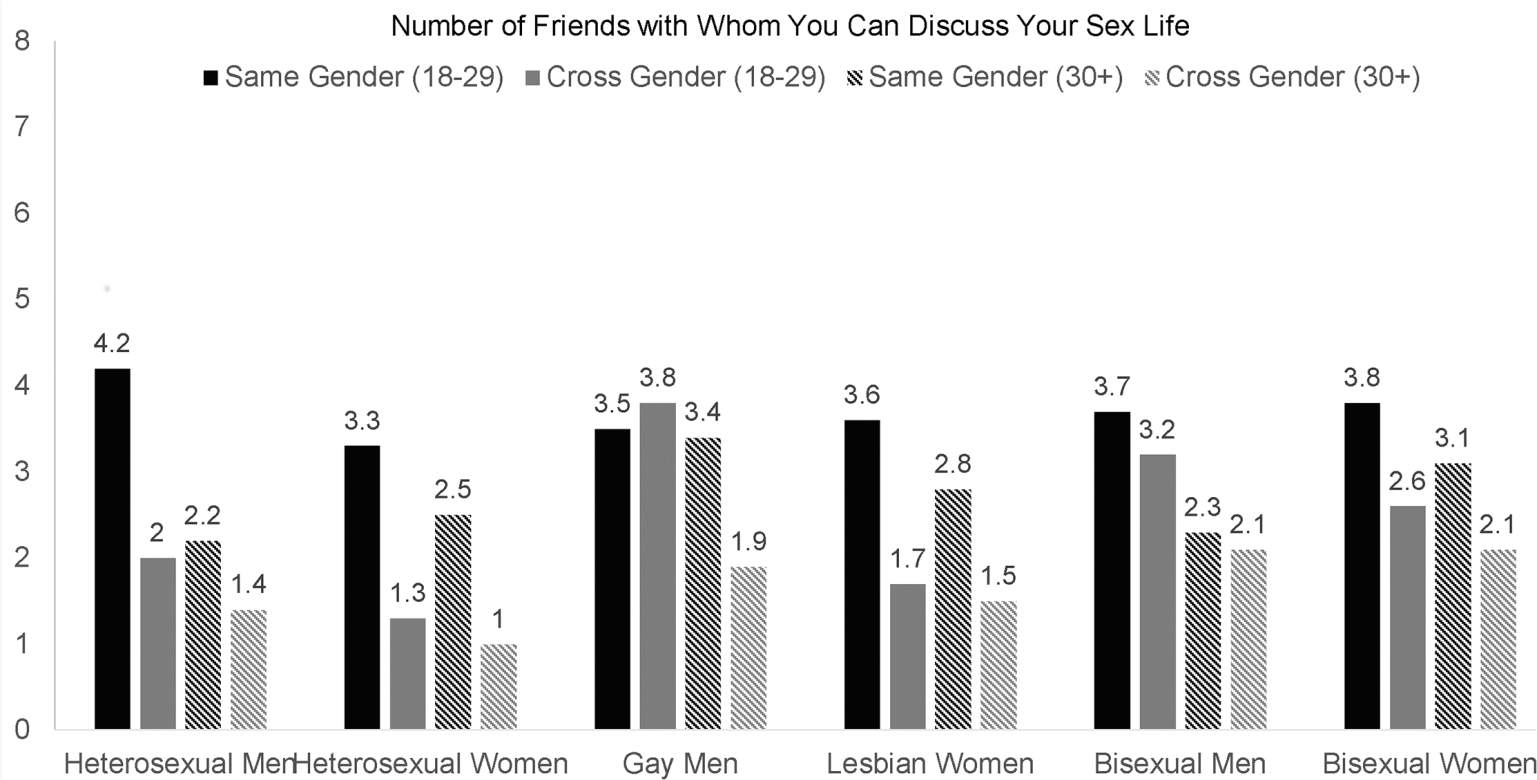
Differences in the Number of Same-Sex and Cross-Sex Friends with Whom Participants Can Discuss Their Sex Lives for Sexual Orientation Groups.

**Fig 2:**
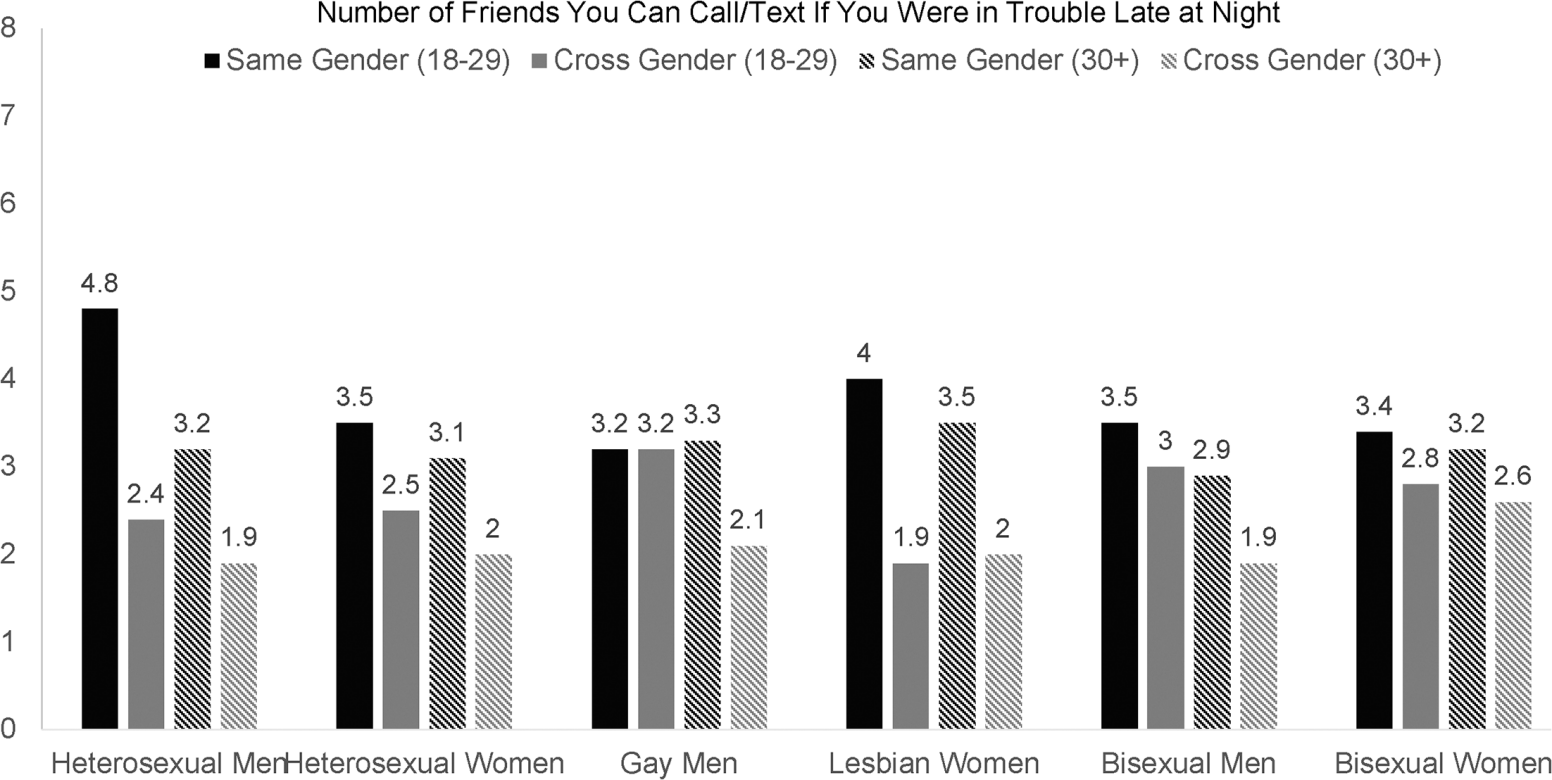
Differences in the Number of Same-Sex and Cross-Sex Friends Participants Can Call/Text If They Were in Trouble Late at Night for Sexual Orientation Groups.

**Fig 3:**
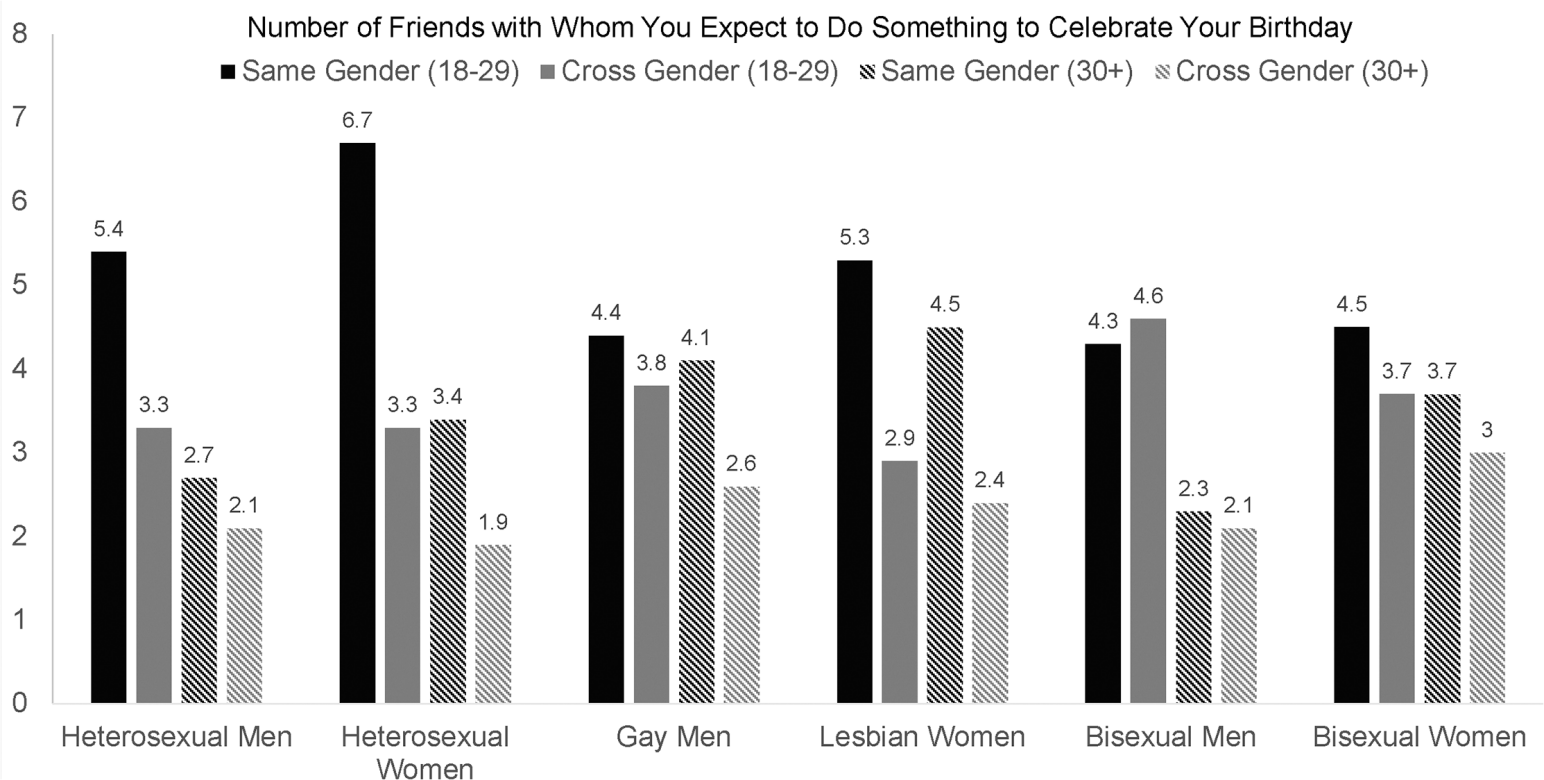
Differences in the Number of Same-Sex and Cross-Sex Friends with Whom Participants Can Expect to Celebrate Their Birthday for Sexual Orientation Groups.

To illustrate further some of the interactions between age, gender, and sexual orientation, we reported the mean number of same-gender and cross-gender friends for young adult (18–29) and older adult (age 30+) participants. These two ranges were chosen because respondents’ number of friends was generally higher among young adults compared to adults, and because there would have been limited statistical power if we divided the GLB individuals into additional age categories. For each group, we show the mean number of same-gender and cross-gender friends with whom participants could discuss their sex lives (Fig 1), could call or text if they are in trouble late at night (Fig 2), and could expect to do something to celebrate their birthday (Fig 3). The magnitude of the difference in number of same-gender vs. cross-gender friendships for each of these groups is shown on the bottom of Table 3.

Looking at the magnitude of mean differences in same-gender vs. cross-gender friends in terms of effect size *d* (Table 3), homophily by gender was common in most groups. The only groups that did not report more same-gender than cross-gender friends were young adult gay men and young adult bisexual men. Adult bisexual men also did not consistently exhibit homophily by gender, reporting more same-gender than cross-gender friends on only one of the three friendship variables. Additionally, although bisexual women did show homophily by gender, the effect sizes were small (*d*s = .19-.33). The findings were partially consistent with the hypothesis that homophily by gender would be less common among gay, lesbian, and bisexual men and women than among heterosexual men and women.

### Hypothesis 3: Friendship and Well-Being

Finally, to test Hypothesis 3, we examined the extent to which number of friends and friendship satisfaction were linked to life satisfaction for each group. We first conducted a regression analysis with the full sample, with follow-up regression analyses for each of the six gender by sexual orientation groups. The VIFs for all analyses were smaller than 5.0, suggesting that multicollinearity was not a substantial problem.

We hypothesized that friendship satisfaction and number of friends would be stronger predictors of life satisfaction for gay men, lesbians, and bisexual men and women than for heterosexual participants. As can be seen in the analysis of the full sample (Table 5), associations between number of friends and life satisfaction were weak (all *βs* < |.04|). Friendship satisfaction and job satisfaction were the two strongest predictors of life satisfaction (*βs* = .29, *p* < .001). There were interactions of friendship satisfaction with gender and with the dummy coded gay/lesbian variable, suggesting that the importance of friendship satisfaction would vary across differing genders and sexual orientations.

**Table 5:** Regression of Friendship Satisfaction and Total Number of Friends on Life Satisfaction (Hypothesis 3).

	Full Sample	Het.Men	Het.Women	Gay Men	Les. Women	Bi.Men	Bi. Women
	*β*	*β*	*β*	*β*	*β*	*β*	*β*
Friendship Variables							
Satisfaction with Friends	.26***	.27***	.25***	.15***	.31***	.35***	.31***
Same-Gender Talk Sex	.00	.00	-.01	.04	-.12	.08	-.01
Cross-Gender Talk Sex	-.02**	-.02	-.02***	.01	.02	-.19**	-.05
Same-Gender Text	.03**	.03*	.02	-.01	.05	.00	.13*
Cross-Gender Text	.02***	.01	.04***	.04	-.07	.02	-.03
Same-Gender Birthday	-.02**	-.04***	-.01	.09	.03	.00	-.06
Cross-Gender Birthday	.03***	.05***	.02	-.06	.15	.18*	.03
Satisfaction Variables							
Job	.29***	.31***	.27***	.40***	.28***	.21***	.24***
Health	.18***	.19***	.18***	.12*	.01	.21***	.17***
Physical Appearance	.15***	.12***	.17***	.13**	.25***	.09	.23***
Demographics							
Age	-.04***	-.05***	-.04***	.03	-.07	.04	-.07
Age^2^ (curvilinear)	.05***	.05***	.06***	.08	-.01	.01	-.02
Education	.01*	-.01	.03***	.02	.11	-.04	.03
Income	.01	.03**	.00	-.03	-.02	.00	-.08*
Children	.03***	.02	.04***	-.09	.03	.05	.03
Marital Status	.11***	.09***	.12***	.16**	.13*	.05	.05
Key Groups of Interest							
Bisexual	-.01	—	—	—	—	—	—
Gay/Lesbian	.00	—	—	—	—	—	—
Gender	.05***	—	—	—	—	—	—
Gender X Bi.	.00	—	—	—	—	—	—
Gender X Gay/Les.	.01	—	—	—	—	—	—
Model Statistics							
*df*	(16,23,545)	(16,11,157)	(16,11,024)	(16,302)	(16,188)	(16,332)	(16,457)
*Adj*. *R* ^*2*^	.41***	.45***	.37***	.43***	.36***	.44***	.44***

*** *p* < .001

** *p* < .01

* *p* < .05.

Separate OLS regressions examining the predictors of life satisfaction are shown for the overall sample and then for each specific gender by sexual orientation grouping. The reference groups for dummy coded variables were: Gender (Men), Gay/Lesbian and Bisexual (Heterosexual), Children (No Children), and Marital Status (Unmarried).

We then conducted regressions with the predictor variables for each gender by sexual orientation grouping. Across all groups, in contrast to our hypothesis, *number of friends* was mostly unassociated with life satisfaction. The only three exceptions (where *βs* were > |.04|) were for bisexual women (call or text if in trouble late at night), and two predictors for bisexual men, where number of cross-gender friends to celebrate one’s birthday and number of cross-gender friends to discuss one’s sex life had strong opposing effects on life satisfaction. Given that these were the only two significant associations, this could have occurred by chance, and thus the finding may simply be a statistical anomaly (VIFs for each predictor were not high, however: cross-gender discuss sex life = 2.94; same-gender celebrate birthday = 3.59).

In contrast, *friendship satisfaction* was strongly associated with life satisfaction for all groups. Consistent with the hypothesis that friendship satisfaction matters most for GLB participants, the strongest overall associations between friendship satisfaction and life satisfaction were for lesbian women, bisexual men, and bisexual women. Tests for significant differences between the slopes, however, did not reveal any statistically significant differences between the beta values for these groups compared to heterosexual men or women (all *p*s > .05).

## Discussion

### Key Findings

#### Sexual orientation differences in friendship patterns

In contrast to the hypothesis that heterosexual participants would report fewer friends than gay men, lesbians, or bisexual participants, regression analyses revealed only small differences when factors that covary with sexual orientation and number of friends were controlled. The largest difference was that gay men and lesbian participants reported fewer same-gender friends than heterosexual participants to celebrate their birthday, but this difference was small (*β* = .07). Although our hypothesis was not supported, our findings highlight the complex nature in which friendship patterns can be assessed. It may be that alternate versions of assessment, such as the perceived quality of those friendships, or the strength of the friendship bond, may have produced findings more in line with our hypothesis.

#### Homophily

Consistent with prior research [35], heterosexual participants reported more same-gender than cross-gender friends across all friendship types. This was unsurprising when considering O’Meara’s identification of the management of sexual attraction as one of the key challenges faced by heterosexual cross-gender friends; avoiding these friendships or keeping them to a minimum may be an effective way of sidestepping unwanted sexual tension [35].

We hypothesized that homophily would be less common among GLB individuals. Results from the regression analyses were not strongly supportive of this hypothesis in terms of overall sexual orientation differences. Looking across different age groups, some findings were consistent with the proposal that homophily would be less common among GLB participants. Young adult gay men and young adult bisexual men showed no evidence of homophily, and adult bisexual men showed homophily in only one of the three aspects of friendship. Although bisexual women displayed homophily, the effect sizes were generally weaker than for heterosexual men and women. The only GLB group to display homophily to the same extent as heterosexual men and women was lesbian women. This is partially in contrast to previous findings, where Galupo [3] concluded that sexual minority individuals displayed similar patterns of homophily by gender (i.e. same-gender friendships) when compared to heterosexuals.

#### Friendship and well-being

Friendship satisfaction was strongly associated with overall life satisfaction for all groups. This result corroborates a large body of research that has shown that friendships satisfy many important needs in our lives: the need to bond with someone like us in some ways and unlike us in others, having someone to call on for comfort in times of turmoil, and someone with whom we can share memorable experiences [11, 12, 36]. In contrast to the hypotheses based on minority stress theory, however, friendship satisfaction was not strongly associated with life satisfaction for GLB participants than for heterosexual participants. Additionally, number of friends was not strongly associated with life satisfaction for any group.

### Limitations and Strengths

Although our sample was large and geographically diverse, it was not probability-based. Internet samples, including ours, tend to include higher-educated and higher-income participants than the U.S. national population, but also tend to be more diverse in gender, age, socioeconomic status, and geographic region than non-probability samples generated by many traditional data-gathering methods [37–38]. Internet surveys can yield a significant advantage over probability-based samples, which typically result in smaller samples overall and therefore limited ability to examine gay, lesbian, and bisexual participants separately. Additionally, these surveys can be completed with ease from the privacy of respondents’ homes or workplaces, thereby reaching individuals who would not otherwise have the opportunity to participate in research studies.

One strength of our study is that we avoided common biases present in previous studies of friendship. In contrast with many previous studies that relied on a single probe taken from a general survey or that focused only on assessing forms of personal disclosure, we also assessed aspects of instrumental and companionate support. Of course, while these distinctions are important, there are still a number of alternative ways to operationalize the different types of friendship, and some of the items may have different meanings to different sets of participants (e.g., the type of “trouble” one might be called on for late at night). Nevertheless, our measurements of specific forms of expressive, instrumental, and companionate friendship may serve as a useful launching pad for future research to create a more detailed taxonomy of friendship and its operationalization.

In addition, we did not know the sexual orientation of participants’ friends. Prior research has found that sexual minority men and women have more cross-orientation friendships when compared to heterosexual men and women [25, 39]. Ueno [40] reported that heterosexual females were more likely than heterosexual males to engage in cross-orientation friendships with sexual minority males, probably because there are fewer problems with sexual tension or romantic interests between the two. Future research assessing sexual orientation of friends will provide a clearer picture of the extent to which homophily occurs by both gender and sexual orientation. Additionally, future research might explore additional correlates of life satisfaction and friendship homophily given the small r^2^ values in the models presented above.

## Conclusion

The role of friendship for gay men, lesbians, and especially bisexual men and women has been understudied. To our knowledge, this is the first study to provide comprehensive comparisons of the same-gender and cross-gender friendship networks of gay men, lesbians, and bisexual and heterosexual men and women. Although comparative studies examining the friendships between these groups have tended to combine gay, lesbian, and bisexual men and women into the singular category of “sexual minorities” [39, 41], our sample was large enough to consider friendship differences across each gender *and* sexual orientation separately.

Overall, our findings suggest that sexual orientation differences in number of same-gender and cross-gender friends are generally small or non-existent, and satisfaction with friends was equally important to overall life satisfaction for all groups. Despite a large body of literature that suggests that gay men and lesbians use their friendships differently [11, 42], we found that there are indeed similarities that should be emphasized. The greater reliance on friends among gay men, lesbians, and bisexual men and women has been true of past cohorts due to historical contexts and more prevalent homophobia—however, because of the shift toward acceptance of GLB individuals, these trends may not persist in present and future cohorts. That said, however, the extent to which people’s friendship patterns demonstrated homophily by gender varied by gender, sexual orientation, and age. Younger gay and bisexual men, and to some extent bisexual women and older bisexual men, did not conform to gendered expectations that people affiliate primarily with their own gender. The similarities in friendship patterns observed by gender and sexual orientation may reflect growing gender egalitarianism and increased social acceptance of GLB individuals throughout the U.S.

## Supporting Information

S1 Dataset (XLSX)
